# Hormone Receptors and Epithelial Ovarian Cancer: Recent Advances in Biology and Treatment Options

**DOI:** 10.3390/biomedicines11082157

**Published:** 2023-08-01

**Authors:** Fulvio Borella, Stefano Fucina, Luca Mangherini, Stefano Cosma, Andrea Roberto Carosso, Jessica Cusato, Paola Cassoni, Luca Bertero, Dionyssios Katsaros, Chiara Benedetto

**Affiliations:** 1 Gynecology and Obstetrics 1U, Departments of Surgical Sciences, City of Health and Science, University of Turin, 10126 Turin, Italy; stefano.fucina@gmail.com (S.F.); stefano.cosma@unito.it (S.C.); andrea88.carosso@gmail.com (A.R.C.); dhocc@libero.it (D.K.); chiara.benedetto@unito.it (C.B.); 2 Pathology Unit, Department of Medical Sciences, University of Turin, 10126 Turin, Italy; luca.mangherini@unito.it (L.M.); paola.cassoni@unito.it (P.C.); luca.bertero@unito.it (L.B.); 3 Laboratory of Clinical Pharmacology and Pharmacogenetics, Department of Medical Sciences, Amedeo di Savoia Hospital, University of Turin, 10149 Turin, Italy; jessica.cusato@unito.it

**Keywords:** ovarian cancer, hormone therapy, endocrine therapy, hormone receptors, aromatase inhibitors, fulvestrant, tamoxifen, letrozole

## Abstract

Epithelial ovarian cancer (EOC) is a significant cause of cancer-related mortality in women. Despite advances in diagnosis and treatment, EOC remains a challenging disease to manage, and the 5-year survival rate is still poor. The role of hormone receptors (HRs) in EOC carcinogenesis and prognosis has been actively explored; however, the role of hormone therapy (HT) in the treatment of these tumors is not well established. Most available data on HT mainly come from retrospective series and small early clinical trials. Several of these studies suggest that HT may have a role in adjuvant, maintenance therapy, or in the case of recurrent disease, especially for some subtypes of EOC (e.g., low-grade serous EOC). Furthermore, HT has recently been combined with targeted therapies, but most studies evaluating these combinations are still ongoing. The main aim of this review is to provide an overview of the progress made in the last decade to characterize the biological and prognostic role of HRs for EOC and the developments in their therapeutic targeting through HT.

## 1. Introduction

Epithelial ovarian cancer (EOC) is the third most common gynecological malignancy worldwide, with 313,959 new cases reported in 2020. It is also one of the deadliest malignancies, with over 200,000 deaths reported globally in the same year [1]. EOC accounts for about 90% of ovarian tumors and comprises several distinct subgroups with different molecular profiles, biological behaviors, and clinical features. Currently, five subgroups of EOC are described: high-grade serous EOC (70%), endometrioid (10%), clear cell (10%), mucinous (3%), and low-grade serous EOC (<5%) [2,3,4]. High-grade serous EOC is characterized by several molecular aberrations and mutations: *TP53* is mutated in almost all cases [5,6,7,8], and somatic or germline mutations of homologous recombination genes such as *BRCA1* and *BRCA2* are also involved in EOC carcinogenesis [5,6]. Moreover, high-grade serous EOC shows widespread accumulation of copy number alterations [7,8], and other pathways involved in high-grade serous EOC are FXM1, Rb1, PI3K, and Notch 1 [5,9]. Clear cell EOC and endometrial EOC share similar patterns of mutations, including alterations in *ARID1A*, *PIK3CA*, *PTEN*, and *KRAS* [10,11]. Several mucinous EOCs have *KRAS* mutations and HER2 amplification [12,13,14], while low-grade serous EOC exhibits activation of the mitogen-activated protein kinase (MAPK) pathway via *NRAS*, *KRAS* or *BRAF* mutations [15,16,17]. EOC has a poor prognosis, with a 5-year relapse rate of 75% for patients diagnosed with advanced disease (International Federation of Gynecology and Obstetrics-FIGO stage III-IV) [15] and a low 5-year overall survival (OS) [12]. To address these poor survival outcomes, better treatment strategies have been developed over the last decade, including optimal debulking surgery to achieve no macroscopic residual tumor and new targeted therapies, particularly for high-grade serous EOC. For instance, bevacizumab, a recombinant humanized monoclonal antibody that blocks angiogenesis by inhibiting vascular endothelial growth factor (VEGF), is approved for maintenance treatment of high-risk EOC [16]. Several trials evaluated the efficacy of inhibitors of the poly ADP ribose polymerase (PARPi) enzyme, and these drugs have been recently approved for treating EOC. Olaparib for *BRCA*-mutated patients as a maintenance treatment after first-line chemotherapy and at platinum-sensitive relapse; Niraparib and Rucaparib at platinum-sensitive relapse regardless of *BRCA* status. PARPis improve progression-free survival (PFS), but, to date, no significant impact on OS has been observed [17,18,19,20,21]. Among emerging target therapies for EOC, several studies have investigated the role of immune checkpoint inhibitors, but with little impact on survival [22,23,24]. This scenario highlights the need to explore alternative treatment options capable of improving survival outcomes without decreasing quality of life. Hormone therapy (HT) is an old but important option with promising results in the maintenance treatment of EOC, especially for the low-grade serous histotype [25,26]. However, the evidence about the efficacy of HT is limited to retrospective studies and small phase II trials. The aims of this review are to summarize the developments in the field of HT in the last decade and offer an overview of the biological and prognostic significance of the hormone receptors (HRs) in EOC.

## 2. The Role of Hormone Receptors in Ovarian Cancer Carcinogenesis

Estrogen Receptor (ER) is a key receptor in the development and progression of EOC. Two different ERs isoforms have been described: ERα and ERβ encoded by *ESR1* (6q25.1) and *ESR2* (14q23.2), respectively. They share a similar structure with other steroid hormone receptors, with an N-terminal domain (NTD), a C-terminal domain corresponding to the DNA binding domain (DBD), a hinge region, and a ligand binding domain (LBD). When the ligand binds to the receptor, the latter forms a dimer and translocates from the cytoplasm to the nucleus, where it acts as a transcription factor (TF), binding to Estrogen Responsive Elements (EREs) [27].

ERα and ERβ expression levels vary in different tissues, and in the ovary, ERβ is more dominant [28]. In preclinical models, ERβ acts as a tumor suppressor and ERα as a pro-tumorigenic factor in breast, prostate, colon, and ovarian cancer cells [27]. Treeck et al. showed that ERβ inhibits cell proliferation by increasing p21 and triggering apoptosis in SK-OV-3 ovarian cancer cells [29]. Furthermore, Bossard et al. showed that ERβ also reduces pro-tumoral factors such as P-AKT, P-RB1, CycD1, and CycA2 in BG-1 (ERα-positive) and PEO14 (ERα-negative) cell lines transfected with *ESR2* adenoviruses. These results were confirmed in mouse models [30].

Additionally, Liu et al. used RNA-seq analysis to show that ERβ can alter the expression of several pro-tumoral genes when activated by an agonist. They also showed that ERβ inhibits NF-kB through a non-canonical interaction with its subunit p65 and that ERβ enhances the sensitivity of chemoresistant EOC cell lines to chemotherapy. Pinton et al. confirmed this effect in naïve EOC cell lines [31,32].

However, not all ERβ isoforms have anti-tumoral effects. Chan et al. transfected EOC cell lines with different ERβ isoforms and found that isoforms -2 and -5 increased the aggressiveness and dissemination of the EOC cells. Specifically, ERβ-5 activated the FAK/Src pathway, which promoted cell migration and proliferation. These effects were reversed by a FAK inhibitor [33]. The loss of ERβ and the imbalance of the ERα/ERβ ratio are crucial for EOCs carcinogenesis, tumor progression, and dissemination, as shown by in vitro experiments [34,35,36]. These findings have been validated in patient-derived samples by immunohistochemical analysis of surgical specimens. A cohort study on 171 EOC patients (mainly represented by the serous histotype: 134/171, 78.36%) at different FIGO stages revealed a higher expression of the inactive cytoplasmic form of ERβ (cERβ) [37], confirming a previous result on a case series of 58 serous EOCs [38]. This finding is supported by a recent study on ERβ performed on TMA samples of EOC, which also reported a correlation between ERβ nuclear/cytoplasmic staining and known clinical risk factors such as the number of pregnancies [39].

ERα activates downstream pathways crucial for carcinogenesis, such as IL6/STAT3, PI3K/AKT, MAPK signaling, and pro-invasive pathways [40]. Additionally, Benhadjeba et al., through in vitro experiments on EOC cell lines, proved a feed-forward mechanism between ERα and the CXCR7/CXCL11 chemokines axis, which activates Erk1/2 and phosphorylates ERα at Ser-118, leading to a more aggressive pro-metastatic tumor phenotype [41]. Moreover, in mice models, Hodgkinson et al. showed the role of GREB1 (Growth Regulation by Estrogen in Breast cancer) in EOC as a promoter of tumor development and growth, being a possible cofactor of ERα in the transcription of ERE genes [42].

Progesterone is another steroid hormone involved in female cancer development by modulating the transcription of several genes. The two main isoforms of progesterone receptor (PR), PRα and PRβ, are encoded by *PGR*, localized on the long arm of chromosome 11 (11q22). PRα and PRβ have the canonical structure of steroid HRs but differ in a 164 amino acid region absent at the N-terminal of PRα, leading to different binding of Progesterone Responsive Elements (PREs) [43].

Progesterone and PR have an anti-tumoral role in ovarian carcinogenesis, unlike their pro-tumoral role in breast cancer [44]. Therefore, the contrasting interaction between the ER and PR in EOC is expected [45]. Mukherjee et al. compared ovarian (OVCA) and breast (MCF-7) cancer cell lines stimulated with estrogen, demonstrating that EOC cells are characterized by an ER-dependent downregulation of *PRG* expression that can be reverted through ER antagonists [46]. Progesterone alone or with estrogen also inhibited the ER-dependent activation of the WNT/β-catenin pathway in EOC—initiating lesions in both human serous EOC cells and murine models [47]. This anti-tumoral ability was also linked to the induction of specific cell death programs such as senescence and necroptosis. Diep et al. observed a strong interaction between FOXO1 and PRβ in vitro in PEO4 cell lines (ERα-positive cells). FOXO1 is a direct interactor of specific steroid HRs, including both PR isoforms [48]. In EOC, PRβ appears to recruit FOXO1 and form a transcriptional complex upon progestin stimulation. This complex enhances the expression of the pro-senescent factor p21 or other senescence effectors (p15, p16, and p27) in case of p21 loss [49,50]. Studies on high-grade serous EOC in murine models, which is characterized by *TP53* loss [51], revealed the importance of progesterone and PR for activating the necroptosis death program, which depends on the TNFα/RIPK1/RIPK3/MLKL pathway [52,53]. PR’s anti-tumoral effects also seem to be related to the induction of A Disintegrin and Metalloproteinase with ThromboSpondin motifs (ADAMTS) proteases [54]. ADAMTS are involved in fertility-related physiological functions of the ovary as well as anti-tumoral effects [55]. ADAMTS1, an inhibitor of the VEGFR pathway, resulted in being directly induced by PR through C/EBPβ, NF1-like factor, and Sp1/3 co-factors in a murine model [56]. According to a recent in vitro study, activation of PR increases both its and ADAMTS4′s expressions; this mechanism seems to be protective against ovarian carcinogenesis. Conversely, PR and ADAMTS losses occur in metastatic cancers [57,58]. Recently the PR anti-tumoral role in the ovary has been questioned since some authors provided data about its relevance in cancer development and quiescence. Wetendorf et al. used transgenic mice to demonstrate that PR overexpression, especially of PRβ, can promote the formation of hormone-dependent ovarian and endometrial neoplasms through the activation of the PI3K-AKT pathway and a cyclin D1-mediated deregulation of the cell cycle [59]. Moreover, Mauro et al., studying p53-mutant fallopian tube epithelial cells transfected (FTE) with PRα or PRβ constructs, assessed the role of PR and progestins in the progression of Serous Tubal Intraepithelial Carcinoma (STIC) into high-grade serous EOC. Through several in vitro experiments, the authors demonstrated different behaviors depending on the presence of progestins; in the absence of progestins, PR+-p53 mutant-FTE cells proliferated due to the inhibition of dual-specificity dimerization partners DP1/2, Rb-like p130/p107, E2F4/5 plus the core complex MuvB (LIN9, LIN37, LIN52, LIN54, RBBP4 proteins)/tyrosine-regulated protein kinases (DREAM/DYRK1) complex, while, on the contrary, in presence of progestins, the DREAM/DYRK1 complex is activated. The latter causes a tumor-quiescent status and promotes PR+ emboli formation, increasing invasive features mainly responsible for the EOC dissemination in the peritoneal cavity [60].

Androgens also exhibit an important role in female physiological and pathological processes. Notably, these hormones can be converted into estrogens by CYP19A1-mediated in situ conversion, thus promoting ER-related cancer growth [61]. Moreover, androgens can also activate their specific receptor, whose gene (*AR*) is localized on chromosome X (Xq11-Xq-12), and induce the transcription of Androgen Responsive Elements (AREs). The expression of androgen receptors (AR) varies among the different EOC histotypes, being higher in serous than in non-serous neoplasms, and ARs have also been shown to enhance cell proliferation. In vitro studies showed a higher number of cells in S-phase, inhibition of p21 and p27 (master regulators of the cell cycle), and the up-regulation of telomerase expression/activity after androgen stimulation, ultimately promoting tumor growth [62,63,64].

AR-dependent cancer growth stimulation is also mediated by the suppression of anti-tumoral pathways. Specifically, in ovarian cancer cells, the activated form of the receptor seems to be capable of sequestering SMAD3, thus leading to a downregulation of the transforming growth factor β (TGFβ) pathway and resulting in pro-tumoral activity [65]. This correlation between activated AR and TGFβ has also been confirmed in other studies [66,67]. SMAD3 is also involved in other cross-talks affecting AR regulation: Kollara et al., exploring the AR interactome, demonstrated that a ligand-independent interaction between AR and VEPH1 inhibits SMAD3 and p-Akt, resulting in a tumor boost caused by higher AR levels [68].

AR can also activate specific cross-talks with pro-tumor pathways by promoting epidermal growth factor receptor (EGFR) signaling and the secretion of the pro-tumoral IL 6 and 8 [69,70].

Furthermore, the oncogenic activity of AR is attributable to its activity as a transcription factor and the formation of molecular complexes with other proteins. GLI3, a Hedgehog-activated transcription factor, has been described as an interactor of AR in both ovarian and breast cancer cell lines, promoting malignancy [71]. Another recently identified interactor of AR is Nanog, a known stem cell phenotype inducer; Ling et al. showed the increase of Nanog, SOX2, and OCT4 expressions after androgen stimulation in EOC cell lines. These findings illustrate the importance of AR in the establishment of a cancer stem cell niche that promotes cancer growth, progression, and dissemination [72].

These data show that steroid hormones play a key role in EOC development through multiple synergic mechanisms, which could be investigated as novel therapeutic targets.

**Figure 1:**
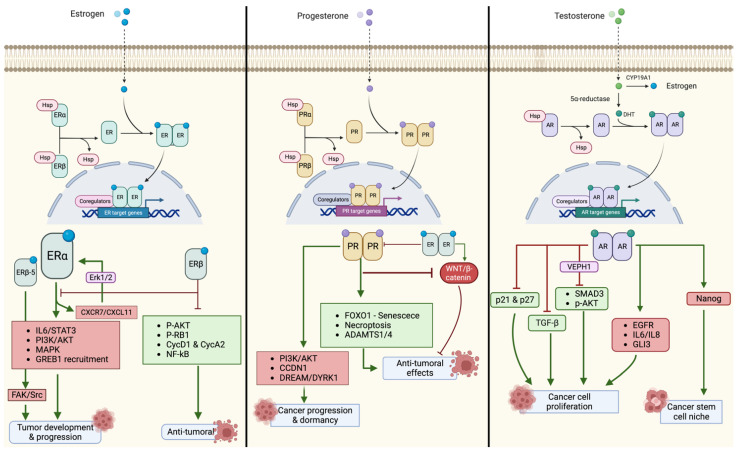
Main pathways activated by hormone receptors and involved in ovarian cancer tumorigenesis.

A summary of the pathways activated by hormone receptors and involved in the pathogenesis of EOC are reported in Figure 1.

## 3. Prognostic Role of Hormone Receptors in EOC

The expression of HRs in EOC has been widely studied as a prognostic factor. Different histotypes of EOC have different expression patterns of HRs. A large analysis of the Ovarian Tissue Analysis Consortium Study on 2933 EOCs showed that high-grade serous, endometrioid, and low-grade serous EOC had strong ER expressions (defined as ≥50% tumor nuclear staining: 60%, 60%, and 71% respectively). In contrast, clear cell and mucinous EOC had low expression of ER (14% and 16%) [73].

ER and PR expressions were also associated with improved survival even after adjusting for age, tumor site, stage, and histological grade at diagnosis [Hazard Ratio: 0.33, 95% Confidence Interval (CI) 0.21–0.51; *p* < 0.0001)]. Moreover, two recent meta-analyses confirmed the favorable prognostic role of ER [74] and PR [75] expression in terms of PFS and OS. ER expression was also associated with less aggressive histological features such as lower lymphovascular space invasion [76], and both ER and PR have also been found to be related to platinum sensitivity [77]. However, a recent meta-analysis questioned these findings, as the use of different antibody clones to determine ER immunohistochemical expression may have influenced the results of previous prognostic studies [78]. On the other hand, the role of AR was controversial. Some studies suggested that women with longer AR CAG repeats had a lower risk of developing EOC, but other authors did not confirm this finding [79]. Similarly, the prognostic impact of AR expression was unclear, as some authors reported a favorable prognostic role, while others obtained inconclusive results [79]. Other studies suggest that low AR expression correlated with a higher risk of developing extra-pelvic metastases, in particular brain metastases [80,81,82,83].

The expression of HRs may correlate with the response to HT, but the evidence is mostly based on retrospective studies [84,85,86]. There is no reliable randomized phase III studies addressing the predictive value of HRs, and this lack of data is due to multiple reasons, including the challenges in defining a meaningful cut-off for stratifying HR expression, the low incidence of some EOC subtypes (e.g., low-grade serous EOC), and the differences in the methods used for their assessment. For example, a recent study suggests that ER immunohistochemistry is not an effective predictive marker of response to HT for low-grade serous EOC. Instead, multigene assays should be used to evaluate ER pathway activation, which could help identify patients who are unlikely to benefit from single-agent HT and those who may need combination therapies [87].

## 4. Aromatase Inhibitors

**Figure 2:**
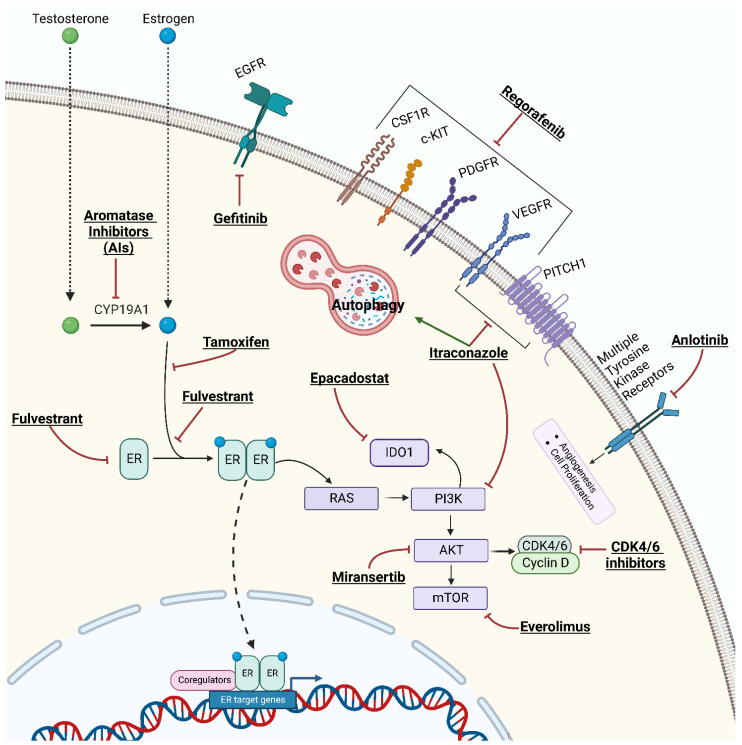
Mechanism of action of hormone therapies and combined target therapies for epithelial ovarian cancer.

Aromatase inhibitors (AIs) (e.g., letrozole, exemestane, and anastrozole) are commonly used HTs for postmenopausal women with ER/PR-positive breast cancer in adjuvant, neoadjuvant, or metastatic settings [88]. AIs block the aromatase enzymes that convert testosterone to estradiol (Figure 2), thereby reducing estrogen production in postmenopausal women [89]. In the 2000s, several studies and trials tested the efficacy of AIs for the treatment of EOC, especially using letrozole and anastrozole, with conflicting results [90]. One of the largest pioneering phase II studies evaluated 60 patients with EOC detected by elevated CA125 levels who received letrozole (2.5 mg daily). Unfortunately, no partial or complete responses were observed by computed tomography in any of the patients, although 10 patients showed disease stabilization for more than 12 weeks [91]. Based on these results, at least seven other phase II clinical trials were conducted between 2003 and 2007 to evaluate the role of AIs in recurrent EOC. The response rates varied from 0% to 38% [92,93,94,95,96,97,98]. Moreover, a recent comprehensive review and meta-analysis of 2490 EOC patients treated with HT reported a summary estimate of a 39% (95% CI 0.29–0.50) clinical benefit ratio for AIs [99]. More recently, a new phase II study (PARAGON—ANZGOG-0903) evaluated the role of anastrozole in 49 women with HR-positive platinum-resistant or refractory recurrent EOC. The study reported a clinical benefit in 13 patients (27%; 95% CI 16–40) despite the absence of complete or partial responses (based on the RECIST criteria). The median PFS was of 2.7 months (95% CI 2.0–2.8 months) [100].

In another phase II study, the PARAGON investigators evaluated the efficacy of anastrozole in 52 patients with asymptomatic HR-positive EOC relapse diagnosed by CA125 elevation. These patients had a low tumor burden and had received only one line of prior chemotherapy. The study reported a 4% complete response and 35% clinical benefit [101]. However, this study has been criticized for its patient selection criteria (e.g., patients with low HR expression levels) [102].

Stanley et al. performed a large retrospective study on 269 patients with relapsed EOC treated with HT, mostly with AIs (77.0% letrozole, 18.6% tamoxifen, 2.2% megestrol acetate, 2.2% other). They investigated the predictive role of HR expression in HT. The CA125 response and clinical benefit rates (response or stable disease) were 8.1% and 40.1%, respectively. The authors also reported that an ER histoscore value > 200 and a time-free interval ≥ 180 days from the last dose of chemotherapy and the initiation of HT were independent predictive factors of response [84].

The role of AIs as first-line maintenance therapy has also been evaluated. In a prospective cohort study on high-grade serous EOC, the addition of letrozole as maintenance was associated with a significantly prolonged recurrence-free survival after 24 months of treatment [60% for letrozole (*n* = 23) vs. 39% for the control (*n* = 27); *p* = 0.035]. A benefit was also observed in patients who received letrozole alongside bevacizumab: 87.5% of patients who received letrozole and bevacizumab had no recurrence after 12 months (*p* = 0.026) [103].

The studies mentioned above involved patients with advanced and/or relapsed high-grade serous EOC; however, several data are also available on the role of HT in low-grade serous EOC. A retrospective study on 64 patients with recurrent low-grade serous EOC evaluated the response to AIs (letrozole *n* = 33 and anastrozole *n* = 21) and tamoxifen (*n* = 17). Among these patients, six complete responses (6.7%, four with letrozole, one with anastrozole, one with tamoxifen) and two partial responses (2.2%, two with letrozole) were obtained. Disease stabilization was reported in 44 patients (33 with AIs and 11 with tamoxifen). These results may be partly explained by the indolent behavior of low-grade serous EOC, but a potential benefit of HT is suggested [104]. Similar results were reported in a phase II study on 36 women affected by HR-positive low-grade serous OC or serous borderline ovarian tumor treated with anastrozole. The study showed a clinical benefit in 61% of patients after 6 months of therapy, and although no patients achieved a complete response, a partial response was reported in 5 patients (14%) and stable disease in 18 patients (50%) [105].

More intriguing results on low-grade serous EOC were obtained when AIs were used in a maintenance setting. A study compared 70 patients with low-grade serous EOC who received maintenance HT (57.2% AIs, 28.6% tamoxifen, 14.2% others) with 133 who underwent observation after primary surgery followed by platinum-based chemotherapy. Median PFS for patients without maintenance HT was 26.4 months, compared with 64.9 months for those who received HT (*p* ≤ 0.001). No statistically significant difference in OS was reported between the two groups (102.7 vs. 115.7 months, respectively) [106]. More encouraging results were reported by Fader et al.: out of 27 patients with low-grade serous EOC treated with HT as maintenance (over 90% with AIs), only 6 patients (22.2%) developed a tumor recurrence, and 2 patients died of disease (after a median follow up of 41 months). Recently, maintenance therapy with AIs for low-grade serous EOC has been analyzed from a cost-effectiveness point of view [107]. The study highlighted that maintenance with letrozole is a cost-effective strategy in women with advanced low-grade serous EOC leading to a clinically-relevant improvement in quality-adjusted life years, life years, and a reduction in the number of recurrences [108].

These results suggest an advantage in terms of PFS in patients with low-grade serous EOC receiving HT.

### 4.1. Combinations Strategies with AIs

Recently, several combinations of target therapy and aromatase inhibitors have been studied to increase the response rate to treatments.

#### 4.1.1. Everolimus and AIs

Oestradiol binds to the ER activating signaling pathways, including PI3K/AKT/mTOR signaling [109]. Small molecules that inhibit the mammalian target of rapamycin (mTOR) kinase activity are being developed to treat various tumors [110,111]. Interestingly, in breast cancer, mTOR inhibitors such as everolimus (Figure 2) can partially overcome AI resistance [112]. A study involving patients with recurrent endometrial cancer found some benefits from treatment with AIs plus everolimus [113]. The PI3K/AKT/mTOR pathway is frequently mutated or activated in EOC and plays a crucial role in tumor progression [114]. In a phase II trial of everolimus and letrozole in relapsed ER-positive high-grade EOC, evaluable patients (*n* = 19) received both oral everolimus (10 mg) and letrozole (2.5 mg orally) daily until disease progression or intolerable toxicity. Three patients (16%) had a confirmed partial response; however, no patient achieved a complete response. Moreover, seven other patients showed a disease control rate of 53%. After 12 weeks of therapy, the PFS was 47%, with a median PFS time of 3.9 months (95% CI, 2.8–11.0) and a 6-month PFS rate of 32% [115].

#### 4.1.2. CDK 4/6 Inhibitors and AIs

Cyclin-dependent kinases (CDKs) are a family of serine–threonine kinases identified in the 1970s–1980s as gene products involved in cell division control. In particular, CDK4 and CDK6 phosphorylate retinoblastoma protein 1 (RB1) and regulate its activity. The active hypophosphorylated form of RB1 acts as a negative regulator of the cell cycle by forming multiprotein complexes that bind the E2F transcription factors and prevent premature cell division. CDK inhibitors (CDKI) inhibit CDK4/6 and lead to hypophosphorylation of RB1 and arrest of cells in the G1 phase (Figure 2) [116,117]. Several molecules (palbociclib, ribociclib, and abemaciclib) have shown significant survival benefits when combined with AIs or fulvestrant in the treatment of metastatic ER-positive breast cancer, leading to their Food and Drug Administration (FDA) approval [118,119]. Interestingly, a significant fraction of EOC showed aberrant expression of cyclins, CDKs, and/or CDKI supporting the hypothesis that these tumors may also respond to CDK4/6 inhibition. Indeed, cyclin inhibitors have been evaluated in different settings for EOC treatment, either as a single agent or in combination with cytotoxic chemotherapy [120].

Based on this background, the association between AIs and cyclin inhibitors has also been evaluated in the treatment of EOC. In a recent trial involving 40 patients with an ER-positive recurrent cancer (20 affected by EOC and 20 by endometrial cancer) treated with 400 mg of oral ribociclib and 2.5 mg of oral letrozole daily, PFS of 50% and 35% were obtained at 12 and 24 weeks, respectively, in the EOC cohort. Interestingly, the greatest benefit was seen in low-grade serous EOC (3 patients were progression-free after 24 weeks of treatment) [121].

#### 4.1.3. Miransertib and AIs

Miransertib is an AKT1 inhibitor (Figure 2) that has been combined with anastrozole in women with PIK3CA and AKT1-mutant ER-positive endometrial cancer and EOC. Preliminary data showed some efficacy in endometrial cancer but not in EOC (data available for 13 patients) [122].

All studies on AIs performed since 2012 are reported in Table 1.

### 4.2. Ongoing Trials

Some trials are currently evaluating the use of AIs for EOC therapy. As mentioned previously, data on AIs are based on retrospective/prospective studies or phase II trials (summarized in Table 2). For the first time, a randomized, double-blind placebo-controlled multi-center phase III trial has been proposed to evaluate the role of letrozole (2.5 mg daily) as maintenance therapy in patients with FIGO Stage II-IV low and high-grade serous or endometrioid EOC [127].

Regarding low-grade serous EOC, a multicenter, randomized, open-label phase III trial is evaluating the superiority of letrozole to conventional carboplatin-taxol chemotherapy (LEPRE trial, NCT05601700). The results of this trial are expected in 2029. Another phase III randomized trial is evaluating letrozole with or without paclitaxel and carboplatin chemotherapy (NCT04095364).

A further phase II study (NCT04720807) is evaluating the combination of anlotinib (a multi-target tyrosine kinase inhibitor targeting tumor angiogenesis and proliferative signaling, Figure 2) and letrozole in patients with relapsed EOC and at least two prior lines of chemotherapy. Currently, 13 patients have been enrolled, and preliminary data showed that 2 and 7 patients achieved a partial response and stable disease, respectively, yielding an objective response rate of 16.7% (2/12, 95% CI: 3.3 to 54.3) [128].

Regarding CDK4/6 inhibitors, an open-label phase II study (NCT04469764) is investigating the efficacy and safety of abemaciclib plus anastrozole or letrozole in patients with hormone receptor-positive OC.

Finally, an interesting study (IMPACT NCT03378297) is evaluating the effect on tumor tissue of four different drugs (acetylsalicylic acid, olaparib, metformin, and letrozole). One of these drugs is taken 10–14 days prior to tumor reductive surgery, starting on the day of laparoscopy.

## 5. Anti-Estrogens

Anti-estrogen agents include a category of drugs that directly interfere with ER signaling and are mainly represented by selective ER modulators (SERMs) and selective ER downregulators (SERDs).

SERMs (e.g., tamoxifen, raloxifene) are anti-estrogen compounds that act as ERα antagonists by competing with estrogen and modulating the transcription of ERα. Tamoxifen is the best-known SERM (Figure 2) which is commonly used for the treatment of premenopausal breast cancer [129]. Currently used SERMs include triphenylethylenes, such as tamoxifen and its analogs; benzothiophenes, such as raloxifene and arzoxifene; phenylindoles, such as bazedoxifene and pipindoxifene; and tetrahydronaphthalenes, such as lasofoxifene. These agents modulate estrogens in breast, bone, and endometrial tissues [130,131]. Differences in the molecular and 3D structures of the co-activators and co-suppressors that modulate the transcriptional activity of the ERs seem to be related to the mechanism of these dual effects that are specific to each tissue [132]. SERDs have antagonistic effects on ERα and ERβ. Fulvestrant is a selective ER degrader that acts by binding, blocking, and degrading the ER, leading to complete inhibition of the estrogen signaling cascade (Figure 2) [133]. Fulvestrant is approved for the treatment of ER-positive metastatic breast cancer alone or in combination with other drugs [133,134].

### 5.1. Tamoxifen

In the last 30 years, several authors investigated the use of tamoxifen in patients with a diagnosis of recurrent or persistent EOC. One of the pioneering studies of anti-estrogen therapy reported three patients with advanced serous EOC treated with tamoxifen: one patient achieved a complete remission lasting for 18 months, and another obtained a partial response. One of the three cases showed high HR expression suggesting a role of this marker in the response to HT [135]. In 1991, Heatch et al. [136] evaluated the response to tamoxifen (40 mg daily) in 105 patients with recurrent or persistent EOC. They found a completed response rate of 10% and a partial response rate of 8%. Furthermore, higher expression of ERs was found in 89% of patients who achieved a complete response and in 59% of patients who achieved a partial response.

The effectiveness of tamoxifen was later reviewed in a Cochrane Systematic Review that included 32 studies. Data from 623 patients were analyzed, of which 60 achieved a partial or complete response (9.6%), and 31.9% showed stable disease. The response rates varied from 0 to 56%, while the no-disease progression rates varied from 0 to 85%. This review did not support a predictive role for HR expression when patients were treated with tamoxifen. The authors concluded that there is only limited evidence of anti-tumor activity based on phase II studies [137]. Another systematic review reported an overall estimated clinical benefit ratio of 43% (95% CI, 0.30–0.56) for tamoxifen [99].

More recently, a retrospective study evaluated tamoxifen in 92 patients with EOC of different stages (I–IV), histotypes (serous, endometroid, clear cell), and clinical settings (first, second, and third line). The clinical benefit ratio was 56%: 10% of patients achieved a partial or complete response, and 46% had stable disease. Also, ER and PR expressions were analyzed in 47 patients, but no correlation was found between clinical response and HR expression or histotype [85].

Ovaresist is a recent phase III trial that compared the efficacy of single-agent chemotherapy (weekly paclitaxel 80 mg/m^2^ or four weekly pegylated liposomal doxorubicin 40 mg/m^2^) and tamoxifen (40 mg daily) in patients with platinum-resistant OC. The primary endpoint was Health-Related Quality of Life (HRQoL), and the secondary endpoints were PFS and OS. Patients (156 and 82) were randomized to chemotherapy and tamoxifen, respectively. Patients treated with tamoxifen had a PFS of 8.3 weeks vs. 12.7 for chemotherapy. OS was not significantly different between the treatment arms. Despite a better PFS in the chemotherapy arm, the patients treated with tamoxifen had fewer side effects and superior HRQoL [123].

Regorafenib is a multi-kinase inhibitor that targets angiogenic (VEGFR1–3, TIE2), stromal (PDGFR-b, FGFR), and oncogenic kinases (KIT, RET, and RAF), as well as tumor immunity (CSF1R) (Figure 2). It is approved for the treatment of refractory metastatic colorectal cancer, unresectable or metastatic gastrointestinal stromal tumors, and hepatocellular carcinoma previously treated with sorafenib [138].

The REGOVAR trial randomized 68 patients to tamoxifen (40 mg daily) or regorafenib (160 or 120 mg daily, 3 weeks on/11 weeks off) until progression or occurrence of toxicity. After a median follow-up of 32 months, there was no difference in PFS and OS between the two groups [124].

Epacadostat is a selective indoleamine 2,3-dioxygenase-1 (IDO1) enzyme inhibitor (Figure 2), currently under investigation in several tumor types [139,140,141]. IDO1 regulates the innate immune response by suppressing T lymphocytes and natural killer cells and by activating regulatory T cells and myeloid-derived suppressor cells [142]. IDO1 also promotes tumor neoangiogenesis through the expression of interferon-γ (IFN-γ) and IL-6 [142,143]. IDO1 is overexpressed in EOC and is associated with advanced stage, chemoresistance, and poor survival [144,145,146].

A randomized, open-label, phase II study [125] compared epacadostat with tamoxifen in biochemically recurrent EOC (CA125 relapse). Forty-two patients were enrolled: the median PFS was 3.75 months for epacadostat (*n* = 22) versus 5.56 months for tamoxifen (*n* = 20, *p* = 0.54). Of evaluable patients, one (5.0%) epacadostat and three (15.8%) tamoxifen patients had confirmed CA125 responses. Despite a supporting preclinical rationale, epacadostat was not superior to tamoxifen in this setting.

The recent studies performed on tamoxifen alone or in combination are summarized in Table 1.

#### 5.1.1. Combination Strategies with Tamoxifen

Tamoxifen has also been investigated in combination with other drugs. AGO-OVAR 2.6 is a phase II trial that investigated the combination of tamoxifen with gefitinib, a signal transduction inhibitor of EGFR tyrosine kinase (Figure 2). However, among 56 patients treated, no survival advantage was observed [126].

#### 5.1.2. Ongoing Trials

Two trials evaluating the use of tamoxifen in combination with other drugs for the treatment of EOC are currently ongoing (reported in Table 2).

The TICTOC study (NCT05156892) is a phase I/II trial investigating the tolerability, toxicity, and efficacy of tamoxifen plus SUBA-itraconazole in platinum-resistant recurrent EOC. Itraconazole is an anti-fungal drug that also has anti-cancer effects by inhibition of angiogenesis, inhibition of the hedgehog pathway, autophagy induction, and reversion of multi-drug resistance (Figure 2) [147,148]. Itraconazole has been tested either as a single agent or with cytotoxic chemotherapy in the treatment of various cancers, including EOC [147]. The TICTOC trial is expected to be completed by 1 January 2025. Another phase II single-arm prospective clinical trial (NCT05669768) is assessing the efficacy and toxicity of the pamiparib + tamoxifen in EOC patients with biochemical recurrence during first-line PARPi maintenance therapy. Pamiparib is a selective PARP1 and PARP2 inhibitor approved in China for the treatment of germline BRCA mutation-associated recurrent advanced ovarian, fallopian tube, or primary peritoneal cancer after two or more lines of chemotherapy [149]. The trial is not currently recruiting patients, and results are expected in 2024.

### 5.2. Fulvestrant

Fulvestrant was investigated in a single phase II study evaluating 26 recurrent EOC patients. Based on CA125 values, one patient obtained a complete response (4%), one had a partial response (4%), and nine had stable disease (35%) [150]. The response to fulvestrant was related to ER and vimentin expression in EOC tissue [86]. More recently, a heavily pretreated patient with ER-positive, recurrent low-grade serous EOC showed a response to fulvestrant and trametinib (a MEK inhibitor) [151].

#### Ongoing Trials

Some trials are testing the efficacy of fulvestrant alone or in combination with other drugs. The FUCHSia Study (NCT03926936) is a phase II trial of fulvestrant in women with ER-positive low-grade gynecological cancers, including EOC. Fulvestrant is also being investigated in combination with the multi-targeted kinase inhibitor regorafenib in a phase II single-arm trial for recurrent low-grade serous EOC (NCT05113368). Another phase II study is evaluating the role of PI3K inhibitor copanlisib in combination with fulvestrant in selected ER-positive and/or PgR-positive advanced EOCs and endometrial cancers with PI3K (PIK3CA, PIK3R1) and/or PTEN mutations (NCT05082025). Ongoing studies investigating fulvestrant for EOC treatment are summarized in Table 2.

## 6. Conclusions

Estrogen, progesterone, and androgens have been studied for their role in EOC carcinogenesis, and literature data suggest that HR-positive EOC have a better prognosis than HR-negative EOC. The currently available studies, mostly retrospective or prospective phase II studies with small sample sizes, have obtained varying and even conflicting results. Nevertheless, HT in EOC could have a similar role to breast cancer, where it is used for adjuvant (first-line), maintenance, and relapse treatment. HT has a relatively low toxicity profile, which makes it suitable for elderly or frail patients who cannot tolerate more aggressive therapies. Moreover, HT seems to prolong the response to chemotherapy and delay disease progression as maintenance therapy. However, more efforts are needed to identify biomarkers that can predict the response to HT and optimize treatment regimens. In this context, some subtypes of EOC, such as low-grade serous EOC, seem to respond better to endocrine therapy. Additional research is needed to ascertain whether combining HT with other drugs, including targeted therapies, can be more effective, and this effort is ongoing. In particular, more multicenter, prospective, well-designed, and randomized clinical trials are warranted to define the role of HT in EOC treatment.

## Figures and Tables

**Table 1:** Main results of studies performed since 2012 exploring the efficacy of hormone therapy for OC.

Ref.	Drug	Study Design	Clinical Setting	Primary Endpoints	Number of Patients Evaluated	CBR	PFS
Bonaventura et al. [100]	Anastrozole	Phase II	Recurrent HR-positive platinum-resistant or refractory OC	CBR	49	39%	2.7 months
Kok et al. [101]	Anastrozole	Phase II	Recurrent asymptomatic HR-positive OC	CBR	52	34.6%	2.7 months
Stanley et al. [84]	Letrozole (77%), tamoxifen, (18.6%), others (4.4%)	Retrospective	Recurrent high-grade serous OC	CBR	269	48.2%	NA
Heinzelmann-Schwarz et al. [103]	Letrozole	Prospective case-control	Maintenance therapy in high-grade serous OC	RFS	50	/	60% after 24 months
Ghersenson et al. [104]	Letrozole (84%), tamoxifen (16%)	Retrospective	Recurrent low-grade serous OC	CBR, PFS, OS	64	9%	7.4 months
Tang et al. [105]	Anastrozole	Phase II	Recurrent HR-positive low-grade serous OC and serous borderline ovarian tumor	CBR	36	61%	9.6 months
Ghersenson et al. [106]	57.2% AIs, 28.6% tamoxifen, 14.2% others	Retrospective-prospective case-control	Maintenance therapy in low-grade serous OC	PFS	203	/	64.9 months
Fader et al. [107]	55.5% letrozole, 37.1% anastrozole, 7.4% tamoxifen	Retrospective	Maintenance therapy in low-grade serous OC	PFS	27	/	79% after 36 months
Colon-otero et al. [115]	Everolimus + letrozole	Phase II	Recurrent ER-positive high-grade serous OC	PFS	19	/	3.9 months
Colon-otero et al. [121]	Ribcoclib + letrozole	Phase II	Recurrent ER-positive high-grade serous OC	PFS	20	/	35% after 24 weeks
Hyman et al. [122]	Miransertib + letrozole	Phase Ib	Recurrent PIK3CA or AKT1-mutant ER-positive OC	Response rate	3	0%	/
Chan et al. [85]	Tamoxifen	Retrospective	All patients with ovarian cancer who received tamoxifen	PFS	92	56%	4.1 months
Lindemann et al. [123]	Weekly paclitaxel or pegylated liposomal doxorubicin vs. tamoxifen	Phase III	Platinum-resistant ovarian cancer	HRQoL	238	/	8.3 weeks
Trédan et al. [124]	Regorafenib vs. Tamoxifen	Phase II	Platinum-sensitive recurrent ovarian cancer with rising CA125 and no evidence of clinical or RECIST progression	PFS	68	/	5.6 month
Kristeleit et al. [125]	Epacadostat vs. tamoxifen	Phase II	Biochemical-only recurrence (CA-125 elevation) following complete remission after first-line chemotherapy	PFS	42	/	5.56 months
Wagner et al. [126]	Gefitinb plus tamoxifen	Phase II	Refractory or resistant to platinum–taxane-based therapy	PFS	56	/	58 days

CBR: clinical benefit rate, ER: estrogen receptor, HR: hormone receptor, AIs: aromatase inhibitors, NA: not available, OC: ovarian cancer, OS: overall survival PFS: progression-free survival, RFS: recurrence-free survival, HRQoL: Health-Related Quality of Life.

**Table 2:** Ongoing trials exploring hormone therapy in OC.

Study	Study Design	Drug	Clinical Setting	Enrollment (Estimated)	Primary Endpoint	Current Status
MATAO; NCT04111978	Randomized double-blind placebo-controlled multi-center phase III trial	Letrozole vs. placebo	Maintenance therapy in low and high-grade ovarian cancer	540 patients	PFS	Recruiting
LEPRE; NCT05601700	Randomized, open-label phase III trial	Letrozole vs. carboplatin + taxol	Adjuvant treatment for low-grade serous OC	132 patients	PFS	Recruiting
NCT04095364	Randomized phase III trial	Letrozole vs. carboplatin + taxol + letrozole	Adjuvant treatment in low-grade serous OC	450 patients	PFS	Recruiting
NCT04720807	Phase II	Letrozole + anlotinib	Platinum-resistant recurrent OC	30 patients	ORR	Recruiting
NCT04469764	Phase II	Letrozole or anastrozole + ademaciclib	Recurrent OC	32 patients	PFS	Recruiting
IMPACT NCT03378297	Phase 0 randomized window-of-opportunity study	Letrozole vs. olaparib, vs. metformin vs. acetylsalicylic acid	Advanced high-grade serous OC before surgery	143 patients	Changes in the expression of biomarkers	Recruiting
TICTOC NCT05156892	Phase I/II	Tamoxifen + SUBA-Itraconazole	Platinum-resistant recurrent OC	44 patients	Recommended phase 2 dose of tamoxifen + SUBA-itraconazole	Recruiting
NCT05669768	Phase II	Tamoxifen + pamiparib	EOC with biochemical recurrence During first-line PARPi maintenance therapy	46 patients	Response rate by CA125	Not recruiting
FUCHSia NCT03926936	Phase II	Fulvestrant	Recurrent/metastatic ER-positive, low-grade gynecological malignancies	200 patients	Response rate	Recruiting
NCT05113368	Phase II	Fulvesrtant + regorafenib	Recurrent low-grade serous OC	31 patients	Response rate	Not recruiting
NCT05082025	Phase II	Fulvestrant + copanlisib	ER+ and/or PR+ ovarian, endometrial breast cancers with PI3K (PIK3CA, PIK3R1) and/or PTEN alterations	78 patients	Safety, tolerability, and dose-limiting toxicities	Recruiting

ORR: objective response rate, OC: ovarian cancer, OS: overall survival PFS: progression-free survival.

## Data Availability

Not applicable.
